# Acute Disseminated Encephalomyelitis Presenting with Neuropsychiatric Symptoms

**DOI:** 10.1155/2024/9810844

**Published:** 2024-09-17

**Authors:** Mrinal Shrestha, Anish Joshi, Ajit Pandey, Aashutosh Chaudhary, Aman Raj Shrestha, Naman Koju, Sujan Timilsina, Ashlesha Chaudhary

**Affiliations:** ^1^ Department of Pediatrics Dhulikhel Hospital, Dhulikhel 45210, Kavre, Nepal; ^2^ Dhulikhel Hospital, Dhulikhel 45210, Kavre, Nepal; ^3^ Department of Radiology Dhulikhel Hospital, Dhulikhel 45210, Kavre, Nepal; ^4^ Everest Hospital Pvt Ltd, New Baneshwor 44600, Kathmandu, Nepal

## Abstract

**Background:**

Acute disseminated encephalomyelitis (ADEM) is a rare immune-mediated pathology involving inflammatory demyelination of the central nervous system. *Case Presentation*. In this case report, we present the case of a nine-year-old female who exhibited altered mental status and focal neurological deficit, subsequently diagnosed as ADEM based on clinical presentation and magnetic resonance imaging (MRI) findings. The patient was managed symptomatically along with glucocorticoids.

**Conclusion:**

ADEM must be suspected when a patient, especially a child, presents with prodromal symptoms followed by multifocal neurological symptoms. Diagnosis can be established with an MRI brain scan. Most patients respond to high-dose intravenous glucocorticoids.

## 1. Introduction

Headache is a common neurological disorder in pediatric patients due to primary and secondary etiologies. A comprehensive headache assessment includes detailed symptom and medical histories, physical examination, and diagnostic evaluations to screen for red flags indicative of more serious secondary disorders, define the headache type(s), and guide overall management [1].

ADEM, also known as postinfectious encephalomyelitis is a demyelinating disease of central etiopathogenesis and is thought to be immune-mediated, because in up to three-fourths of the cases; it follows an antecedent infection or immunization [2]. It is a rare pathology with an average incidence of 0.07 to 0.6 in different population-based studies [3, 4]. The illness usually appears as a single-phase condition with symptoms of encephalopathy and multiple lesions in the brain and spinal cord. Patients can present with altered mental status, focal neurological symptoms, and meningism [4].

The immune system is activated through both humoral and cell-mediated responses, triggered by the resemblance between microbial epitopes and myelin antigens, particularly myelin oligodendrocyte glycoprotein (MOG). This molecular mimicry is thought to be the primary cause of immune-mediated damage [5]. Currently, there are no specific biomarkers to diagnose ADEM, so the diagnosis is based on excluding clinical and laboratory findings, as well as suggestive neuroradiological features of other diseases [6]. ADEM is a self-limiting rare disease and the patient is often given supportive treatment along with specific therapy like glucocorticoids, intravenous immunoglobulin, plasma paresis, and rehabilitation [7].

In this case report, we present a case of a nine-year-old female who presented with complaints of altered mental status and focal neurological deficit, diagnosed as ADEM based on the clinical picture and MRI findings, and the patient was managed symptomatically along with glucocorticoids. This report is written in line with the CARE guidelines [8].

## 2. Case Details

We report a case of a nine-year-old female who presented with a complaint of headache lasting for a day which occurred 13 days back. This was associated with 3 episodes of nonprojectile vomiting. On the second day of illness, she developed abnormal behavior in the form of prolonged staring at inanimate objects and bruxism. Later on, there was an abrupt loss of tone and loss of consciousness lasting for 2-3 minutes. As the days passed, her parents noticed that she was less interactive and she had aggressive outbursts, visual hallucinations, choreiform movements, and hemiballismus.

There was no history of fever, generalized tonic-clonic movement, deviation of mouth, frothing of saliva, uprolling of eye, tongue bite, cough, shortness of breath, palpitation, bluish discoloration of body, loose stool and blood in stool, abdominal pain, abdominal distension burning micturition, decreased urine output or altered color of urine, sore throat, facial puffiness, orthopnea, and palpitations during the entire course of illness. Relevant history revealed a history of fever with chills, body aches, weakness, and retro-orbital pain with a rash all over the body which occurred 2 months back. The illness was resolved with conservative management at home after 3 days. The patient was immunized as per the national immunization schedule [9]. Coronavirus Disease of 2019 (COVID-19) vaccine (2 doses of Vero cell vaccine) was administered. The last dose of COVID-19 vaccination was administered 4 months back. There was a similar history of fever with body aches, rash, and headache 2 months back in all the family members.

For the above complaints, the child was taken to traditional healers but the symptoms did not subside so she was brought to the Emergency Room (ER) of our tertiary care center. In the ER, the Glasgow Coma Scale was 11/15 (E4V2M5), and pupils were 3 mm in size, bilaterally equally reactive to light. The vitals were stable, and random blood sugar was taken which was 112 mg/dl. The child was disoriented to time, place, and person. On motor examination, the tone was increased with power 3/5 in all four limbs with bilateral hyperreflexia and normal plantar response. Signs of meningeal irritation were absent and cranial nerves examination was grossly intact. Her other examinations were also normal.

Per the clinical history and examination findings, the child was admitted to the pediatric intensive care unit. Relevant investigations were sent. A noncontrast computed tomography (CT) scan of the head showed no abnormalities ruling out any gross vascular and neoplastic conditions and a lumbar puncture was performed which revealed normal cerebrospinal fluid (CSF) analysis reports ruling out any infectious cause. The CSF antibodies could not be sent due to financial constraints. The complete blood counts, renal function test, liver enzymes, thyroid function test, electrolytes, and antistreptolysin O titer were within normal range.

**Figure 1:**
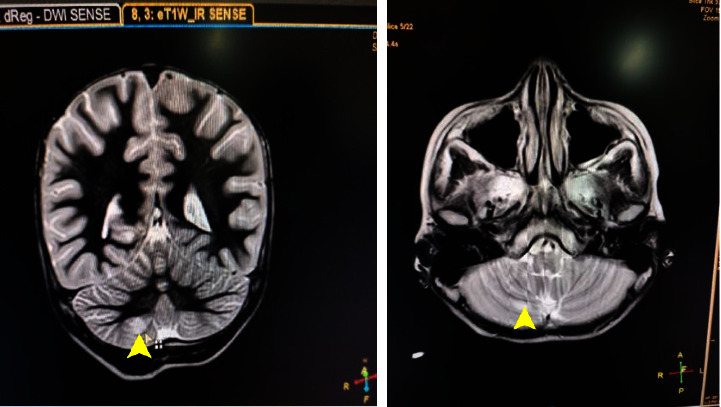
Subtle patchy areas (yellow arrow) of T2-FLAIR hyperintensities noted in inferomedial aspects of the right cerebellar hemisphere.

The rapid development of symptoms with multifocal CNS involvement and no clear infectious etiology strongly suggests a demyelinating process. ADEM was suspected based on clinical presentation, and neurological examination, and MRI was warranted for exclusion of other diagnoses. The MRI findings, along with clinical history, support the diagnosis of ADEM, allowing for appropriate management. On evaluation with contrast-enhanced MRI Brain, subtle patchy areas of T2-weighted-Fluid-Attenuated Inversion Recovery (T2-FLAIR) hyperintensities were noted involving the inferomedial aspects of the right cerebellar hemisphere (Figure 1).

MRI of the spine was also done which was normal. However, the CSF oligoclonal bands, anti-NMDR antibodies, and MOG1 Ab in CSF and serum could not be sent. Based on history, clinical examination, and neuroimaging findings as per the MRI Brain, the diagnosis of ADEM was confirmed despite the unavailability of CSF antibody reports.

Intravenous (IV) methylprednisolone was started at 30 mg/kg/day for 5 days and then tapered gradually to oral dexamethasone. For the abnormal movements, oral phenobarbitone was started along with IV haloperidol. The child's condition improved within 3 days of steroid usage. After 16 days of hospital stay, she was discharged with oral dexamethasone. She was able to speak a few words and ambulation was possible. The child presented after 2 weeks in our OPD. The GCS was 15/15 and the child was able to communicate in the form of short sentences. She was able to walk properly without support.

## 3. Discussion

Early recognition and appropriate interpretation of common neurological symptoms are challenging for healthcare professionals. However, these could also be early symptoms of more serious neurological syndromes [10]. The incidence of delayed diagnosis ranges from 5% to 20% [11] and this lack of early recognition and diagnosis can have life-threatening consequences [12].

ADEM often follows infection or immunization; however, the etiology of ADEM can be difficult to establish in 15–50% of cases [2]. Viruses often associated with ADEM are mumps, rubella, measles, Epstein–Barr virus, and dengue, and bacteria associated with ADEM are *Legionella pneumophila, Borrelia burgdorferi,* and *Mycoplasma pneumoniae* [13]. In our case, the patient had a history of fever with chills, body aches, weakness, and retro-orbital pain with a rash all over the body, 2-3 months back which resolved in 3-4 days of rest and conservative management at home. Since the patient did not seek health care, we could not pinpoint the preceding infection in our case; however, during this time, there was an epidemic of dengue and the patient had a history of symptoms that could be due to dengue virus. Our patient also had taken the COVID-19 vaccine, but the patient had the clinical manifestation of ADEM only after 4 months, making the vaccine a less likely cause.

Diagnosis of acute disseminated encephalomyelitis (ADEM) can be challenging as there are no definitive diagnostic markers for the condition. This highlights the need to recognize ADEM clinically. ADEM can present at any age; however, it is more common in childhood between ages 5 and 8 years, where there is a higher incidence of exanthematous infections and immunization in this age group [7, 13]. It is slightly more common among male children [3, 4]. Clinical features of ADEM can manifest 3–6 weeks after antecedent infection or immunization and the onset can be abrupt or gradual over a few days. Prodromal features like fever, nausea, vomiting, headache, and malaise usually precede neurological symptoms by 2–5 days [13]. ADEM can involve any part of the neuraxis so neurologically there can be a wide range of clinical presentations [13]. Patients can present with multiple symptoms like lethargy, altered consciousness, confusion, psychosis, coma, brainstem syndromes, optic neuritis, transverse myelitis, meningism, dystonia, seizures, and focal neurological deficit [4, 13]. In our case, the patient had multiple neurological symptoms including altered consciousness, bruxism, loss of consciousness, behavioral disorder, visual hallucinations, choreiform movements, and hemiballismus.

International Pediatric Multiple Sclerosis Study Group (IPMSSG) has stated that after the exclusion of similar conditions, diagnosis of ADEM can be met when a patient has a first multifocal clinical CNS event of presumed inflammatory demyelinating cause; with encephalopathy and MRI brain findings in the acute state (3 months phase) with no new clinical or MRI findings 3 months or more after the clinical onset [14]. The best diagnostic tool to evaluate ADEM is MRI. Multifocal hyperintense lesions are demonstrated in the brain in T2 weighted and FLAIR sequences with cotton ball lesions and fuzzy margins. The lesions can involve white matter, gray matter, and the white matter-gray matter junction [15].

The pathology is most effectively revealed by T2-FLAIR sequences, displaying multiple patchy white matter hyperintensities involving the cerebellum and brainstem. Children are more likely to show cerebellar and brainstem involvement. Though white matter is primarily affected, gray matter involvement, specifically in the basal ganglia, thalamus, and brainstem, can also be observed. Some MRI lesions may enhance after gadolinium administration, but this was not the case in our situation. Involvement of the thalamus and sparing of the corpus callosum suggests a higher likelihood of an ADEM diagnosis while simultaneously ruling out Multiple Sclerosis (MS) as a potential diagnosis [16]. In our case, subtle patchy areas of T2-FLAIR hyperintensities were noted involving inferomedial aspects of the right cerebellar hemisphere suggestive of ADEM.

Treatment of ADEM is supportive involving management of the airway, breathing, and circulation with medical therapy which can include glucocorticoids, IV immunoglobulins, and plasma exchange [7]. In the current scenario, high-dose IV corticosteroid is regarded as the first-line treatment for ADEM with full recovery reported in 60–70% of patients [17]. In our case, the patient was given IV methylprednisolone at 30 mg/kg/day for 5 days and then tapered gradually to oral dexamethasone. The recommended dose of steroid therapy consists of IV methylprednisolone 20−30 mg/kg/day over five days followed by oral prednisolone 1-2 mg/kg/day tapered over 4–6 weeks [7, 17]. In patients with contraindications to steroid therapy or those with steroid-unresponsive ADEM, IV immunoglobulins can be a second-line therapy [18]. Fulminant ADEM, refractory to steroids, can be treated with plasma therapy [19].

## 4. Conclusion

ADEM must be suspected when a patient, especially a child, presents with prodromal symptoms, followed by multifocal neurological symptoms. In patients with suspected ADEM, diagnosis can be established with an MRI brain. Most patients respond to high-dose IV glucocorticoids with a high rate of full recovery.

ADEM diagnosis hinges primarily on clinical and imaging results. Identifying ADEM early and accurately is crucial for timely treatment and minimizing neurological harm. Starting the right treatment promptly is key to positive outcomes. Comprehensive care and optimal recovery require a multidisciplinary approach.

## Data Availability

All data underlying the results are available as part of the article and no additional source data are required.
